# Posterior mediastinal ectopic meningioma: a case report

**DOI:** 10.1186/s12957-015-0581-y

**Published:** 2015-04-22

**Authors:** Chenying Lu, Xianghua Hu, Min Xu, Weibo Mao, Hongyuan Yang, Zufei Wang, Jiansong Ji

**Affiliations:** Department of Radiology, Lishui Hospital of Zhejiang University, Lishui Central Hospital, the Fifth Affiliated Hospital of Wenzhou Medical University, No. 289, Kuocang Rd., Lishui, District 323000 Zhejiang China; Department of Pathology, Lishui Hospital of Zhejiang University, Lishui Central Hospital, the Fifth Affiliated Hospital of Wenzhou Medical University, No. 289, Kuocang Rd., Lishui, District 323000 Zhejiang China

**Keywords:** Mediastinum, Ectopic, Meningioma

## Abstract

Primary ectopic meningiomas occurring in the mediastinal region are extremely rare. So far, only five cases of primary mediastinal meningioma have been reported in the literatures. The imaging characteristics and the clinicopathological significance of mediastinal psammomatous meningioma have not been detailed. Here, we report the case of a 42-year-old male with primary posterior mediastinal psammomatous meningioma. The clinical features, imaging, and pathological findings are carefully analyzed, and the relevant literatures were reviewed.

## Background

Derived from the meningothelial cells capping the arachniod villi, meningioma is a common neoplasm of the central nervous system (CNS) [1]. Most meningiomas are located in the cranial cavity, in the vicinity of venous sinus and attached to the dura. Occasionally, primary meningiomas occur ectopically, for example, in the head and neck region (orbit of the eye, nose, paranasal sinuses, mandible, and ear) or paraspinal soft tissues [1-4]. Although exceedingly rare, primary ectopic meningiomas have been reported to occur on the sites including the foot, skin, retroperitoneum, and mediastinum [2,5-7]. The rarity and uncertainty of etiology of primary ectopic meningiomas at these sites may pose difficulties for the correct diagnosis [6,7]. Here, we report the imaging findings and the pathological manifestations of a rare case of primary psammomatous meningioma that occurred in the posterior mediastinal region. The contributions of imaging and pathological examinations to the diagnosis and differential diagnosis are discussed.

## Case presentation

**Figure 1:**
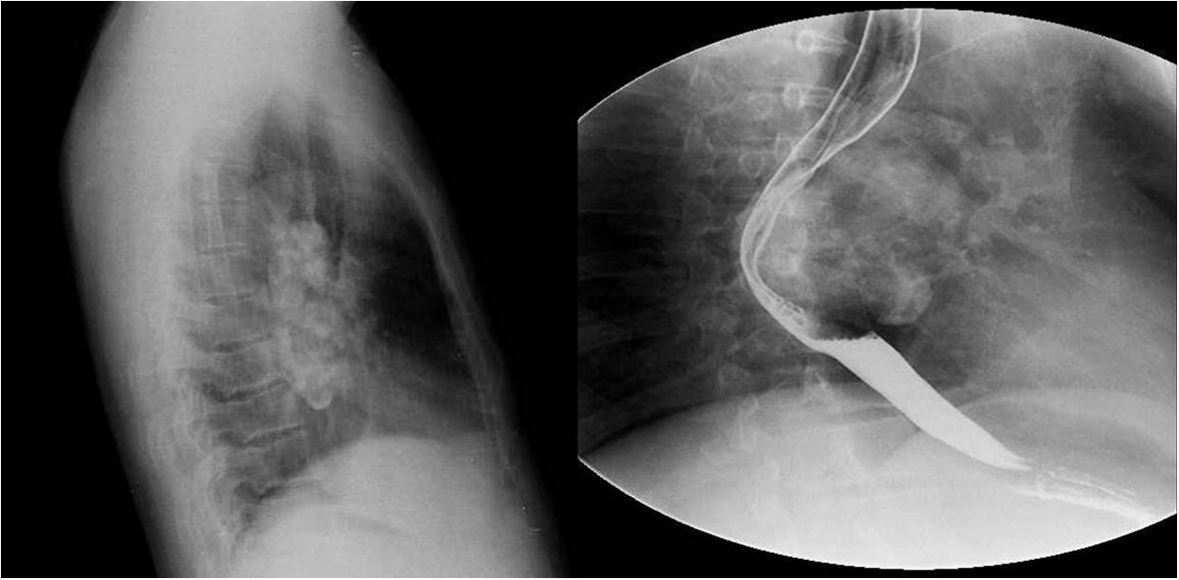
A chest lateral radiograph shows a large mass with lots of calcification in the mediastinum; esophagus barium opacification shows that the middle and inferior segment of the esophagus was compressed by the mass.

**Figure 2:**
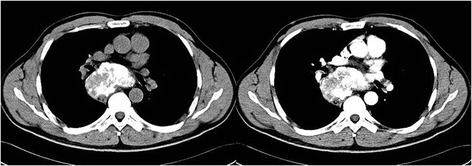
Chest unenhanced CT scan shows a heterogeneous mass with calcification located in the posterior mediastinum. Its border was clearly demarcated, and enhanced CT scan shows the mass with little enhancement.

**Figure 3:**
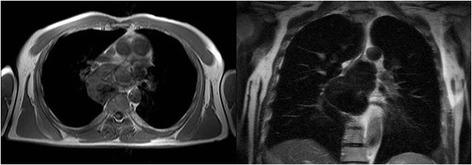
Axial T1-weighted MRI shows a well-circumscribed isointense-hypointense mass located in the posterior mediastinum, and coronal T2-weighted MRI shows a hypointense tumor located under the tracheal carina.

A 42-year-old Chinese male was admitted for a 6-year history of dysphagia lusoria. His past medical history was unremarkable. At admission, general conditions were satisfactory, and a physical examination of the thorax was normal. Routine laboratory tests were within normal values, but a chest radiograph revealed a large mass with calcification in the mediastinum. Esophagus barium opacification showed that the middle and inferior segment of the esophagus was compressed by the mass (Figure 1). A computed tomography (CT) scan of the chest revealed the presence of an ovoid mass about 4.8 × 7.6 × 10.0 cm in the right posterior mediastinal region. The mass showed a heterogeneous density accompanied with pronounced calcification. Administration of contrast medium gave only a mild strengthening of mass (Figure 2). A magnetic resonance (MR) imaging showed a well-circumscribed mass which was isointense-hypointense on T1-weighted imaging and hypointense on T2-weighted imaging (Figure 3). An enhanced MR imaging of the head and spine revealed no abnormal findings. A diagnosis of teratoma was first suggested. The tumor was then surgically removed through a right thorax incision.

**Figure 4:**
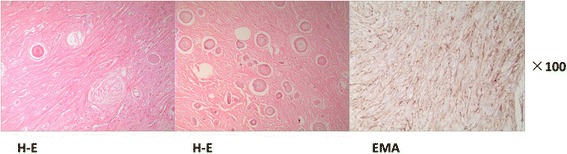
Hematoxylin and eosin (H-E) staining shows that the tumor is composed prominently of elongated spindle cells and collagen fibers; numerous typical whorl formations and multiple psammoma bodies are observed in the tumor. Immunohistochemistry shows positive staining for epithelial membrane antigen (EMA). (Magnification shown at × 100).

The patient underwent surgical resection of the mediastinal tumor through the right thorax incision. During the operation, the tumor was located in the posterior mediastinum. It was found to have extended under the bronchial bifurcation. Based on this surgical finding, the mass was in close relationship with the esophagus and pericardium; the esophagus was obviously compressed and displaced. The tumor was a clearly demarcated encapsulated solid mass with brown-gray color and off-white cut surface. After being routinely fixed with 10% formaldehyde and embedded in paraffin, the tumor sample was processed, and 4-μm-thick tissue sections were obtained. The hematoxylin and eosin (H-E) staining (Figure 4) showed that the tumor was composed predominantly of bundles of elongated spindle-shaped cells with little oval nuclei. There was heavy deposition of collagen fibers between the tumor cells. In some areas, typical whorl structure of mengingioma was observed. In addition, multiple psammoma bodies were seen. There were no mitotic figures and necrosis. Immunohistochemical staining revealed that the tumor cells were positive for epithelial membrane antigen (EMA) and vimentin, and negative for CD34, bcl-2, SMA, and S-100 protein. The Ki-67-labelling index was <5%. According to the histological and immunohistochemical features, the tumor was diagnosed as a benign mediastinal psammomatous meningioma (World Health Organization (WHO) grade I, based on the 2007 WHO classification [1]). At follow-up 24 months after surgery, there was no evidence of local recurrence or distal metastasis.

## Discussion

Meningiomas are usually clinically indolent and morphologically heterogeneous tumors; they may exhibit a variety of different histologic patterns [1,2]. Primary ectopic meningiomas are very rare with the incidence ranging from 0.9% to 2.0% of all meningiomas [8]. Their occurrence in the mediastinum is even rarer [6,7]. Their histopathogenesis is unclear. However, four hypotheses of the formation of ectopic meningiomas have been suggested: (1) direct extension of an intracranial lesion, (2) distant metastasis from an intracranial meningioma, (3) origination from arachnoid cells within the sheaths of cranial nerves, and (4) origination from embryonic nests of arachnoid cells [9].

Imaging features of ectopic meningioma are varied among different histopathogenical types. Fibrous meningioma, malignant meningioma, and angioblastic meningioma located in the mediastinum had been reported in the previous literature [6,7]. The case we report here is a posterior mediastinal psammomatous meningioma with characteristic imaging findings of calcification in the tumor. Imaging findings show a well-defined mass located in the posterior mediastinum with multiple calcification foci, which obviously compresses the esophagus. Dynamic CT only slightly enhanced the mass. These imaging characteristics are similar to those of intracranial- and extracranial-originated calcified meningiomas [1,2]. The imaging differential diagnosis for a posterior mediastinal mass containing calcification foci commonly includes Castleman disease, teratoma, or neurogenic tumor [10-13]. The mass of Castleman disease usually displays punctuate or arborizing calcification with obvious strengthening on an enhanced scan [10,11]. Teratoma often showed heterogeneous densities within the mass caused by calcification and fat tissues [12]. Finally, the most common cause of a posterior mediastinal mass, neurogenic tumors, are often accompanied by cystic or hemorrhagic areas [13].

A definite diagnosis can only be made upon histopathological and immunohistochemical findings. In this case, H-E staining showed that the tumor is composed of elongated spindle cells and collagen fibers, the common features of meningioma [1-9]. The findings of typical whorl formations and multiple psammoma bodies confirm the diagnosis of psammomatous meningioma [1,2]. Immunohistochemistry is a powerful tool to differentiate meningioma from other mesenchymal tumors, such as solitary fibrous tumor, leiomyoma, hemangiopericytoma, and neurogenic tumors. The immunohistochemical staining features of these tumors are summarized in Table 1 [14-19]. The tumor cells in our report showed positive staining for vimentin and EMA, which further support the diagnosis of meningioma [19]. Last, but not least, as there was no clinical or radiological evidence of any intracranial or intraspinal lesion, we concluded that the meningioma in this case was of complete mediastinal origin.

**Table 1:** **Immunohistochemical findings of different mesenchymal tumors**

	**Meningioma** [1 **-** 9 **,** 14]	**Solitary fibrous tumor** [15]	**Leiomyoma** [16]	**Hemangiopericytoma** [14 **,** 17]	**Neurogenic tumors** [18]
Vimentin	+	+	+	+	+
EMA	+	-	-	-	-
CD34	-	+	-	+	-
Bcl-2	-	+	-	+	-
SMA	-	-	+	-	-
S-100 protein	-	-	-	-	+

EMA, epithelial membrane antigen; SMA, smooth muscle actin.

## Conclusions

In summary, primary meningioma of the posterior mediastinum is extremely rare; they need to be differentiated from other tumor masses. Radiological imaging is useful in preoperative diagnosis and surgical planning, but the ultimate diagnosis has to be confirmed by histopathological examination.

## Consent

Written informed consent was obtained from the patient for publication of this case report and any accompanying images. A copy of the written consent is available for review by the Editor-in-Chief of this journal. This case report was performed in compliance with the Helsinki Declaration.

## Abbreviations

CNS: central nervous system
CT: computed tomography
EMA: epithelial membrane antigen
H-E: hematoxylin and eosin
MR: magnetic resonance
WHO: World Health Organization
